# Dynamics of Early Signalling Events during Fracture Healing and Potential Serum Biomarkers of Fracture Non-Union in Humans

**DOI:** 10.3390/jcm9020492

**Published:** 2020-02-11

**Authors:** Agata N. Burska, Peter V. Giannoudis, Boon Hiang Tan, Dragos Ilas, Elena Jones, Frederique Ponchel

**Affiliations:** 1 Leeds Institute of Rheumatic and Musculoskeletal Medicine, University of Leeds, Leeds LS2 9JT, UK; A.N.Burska@leeds.ac.uk (A.N.B.); hiangboon@gmail.com (B.H.T.); D.Ilas@leeds.ac.uk (D.I.); e.jones@leeds.ac.uk (E.J.); F.Ponchel@leeds.ac.uk (F.P.); 2 Leeds Biomedical Research Centre, Leeds Teaching Hospitals NHS Trust (LTHT), Leeds LS9 7TF, UK

**Keywords:** fracture healing, inflammation, signalling

## Abstract

To characterise the dynamic of events during the early phases of fracture repair in humans, we investigated molecular events using gene expression profiling of bone fragments from the fracture site at different time points after trauma and immune/stromal cells recruitment at the fracture site using flow cytometry. Bone and inflammatory markers were expressed at low levels at homeostasis, while transcripts for bone constituent proteins were consistently detected at higher levels. Early after fracture (range 2–4 days), increased expression of CXCL12, suggested recruitment of immune cells associated with a change in the balance of degradation enzymes and their inhibitors. At intermediate time after fracture (4–8 days), we observed high expression of inflammatory cytokines (IL1-beta, IL6), CCL2, the T-cell activation marker CD69. Late after fracture (8–14 days), high expression of factors co-operating towards the regulation of bone turnover was detected. We identified potential soluble factors and explored circulating levels in patients for whom a union/non-union (U/NU) outcome was known. This showed a clear difference for PlGF (*p* = 0.003) at day 1. These findings can inform future studies further investigating the cascade of molecular events following fractures and for the prediction of fracture non-union.

## 1. Introduction

Modern clinical fracture management aims to achieve bone healing in the shortest time frame, with the best possible functional recovery and fewer complications. Fracture healing is a unique and very effective process including complex and well-coordinated interactions among cells, cytokines, bone matrix and mechanical forces [1]. The regenerative process involves several consecutive steps characterised by an inflammatory phase, formation of soft callus, followed by hardening of this callus, and finally remodelling [2]. Successful fracture healing can be defined by adequate callus mineralization and the restoration of biomechanical and functional properties [3].

Delayed bone healing and non-union fortunately is an infrequent event occurring in approximately 5%−10% of long bone fracture cases [4]. Fracture non-union represents a serious health care burden for society, the National Health System (NHS) and patients. Prolonged non-union treatment is associated with significant loss of working days and economic effects and also carries the risk of permanent disabilities related to a dysfunctional limb, joint stiffness, muscular atrophy or reflex sympathetic dystrophy [5]. Cases where fractures fail to heal frequently result in further complications, additional surgery and may be even fatal [4].

There are two distinct variants of non-union with opposed underlying pathomechanisms, hypertrophic and atrophic non-unions [6]. The hypertrophic non-union results from insufficient mechanical stabilisation of the fracture; in contrast, atrophic non-union is a condition where bone fails to heal due to an insufficient biological response at the fracture site (sometimes in combination with mechanical factors). This biological impairment is usually due to insufficiency of essential mediators, or other crucial biological parameters [6]. Nonetheless, the risk factors for atrophic non-union remain obscure.

The role of the immune system in the different stages of fracture repair is still under investigation. However, inflammation is the first essential step in the process. Osteo-immunology, which focuses on the relationships between the immune system and the skeleton, has only recently emerged [7]. It is currently believed that the physiological process of fracture healing begins with a prompt inflammatory response from innate cells (mostly neutrophils) [8,9,10,11] which results in the production of several cytokines and chemokines that in turn stimulate and attract various other cell types to the fracture site (i.e., adaptive immune cells and osteo-progenitors/mesenchymal stem cells (MSCs) [10,12,13]. Adaptive cells such as T and B lymphocytes are critical in the fracture healing course, possibly via T-cell produced RANKL [14], and the interplay between Th17/regulatory T-cell and osteoclasts [15], and the production of pro/anti-inflammatory cytokines [16]. Therefore, a well-controlled balance of inflammatory mediators is essential for appropriate fracture repair [17,18].

Experimental studies have been the mainstay of gathering knowledge of bone healing at the molecular level [2]. The molecular signature of the local environment is still vague due to the complexity and cross talk of the different pathways involved. Moreover, one crucial limitation of the findings of experimental studies remains the inherent differences that exist between the genetic constitution of small animal models and humans [19].

Taking into consideration all of the above, three objectives were set for the herein study. Firstly, to characterise molecular signalling events during the early phases of fracture repair in humans, by determining the gene expression profiling of bone fragments from the site of fracture at different time points after injury. Secondly, to investigate the dynamics of immune cells recruitment at the fracture site. Thirdly, to select potential candidate predictors of an impaired fracture healing response and to test them in a different group of patients with long bone fractures.

## 2. Materials and Methods

### 2.1. Patients for Bone Fragment Samples

**Figure 1:**
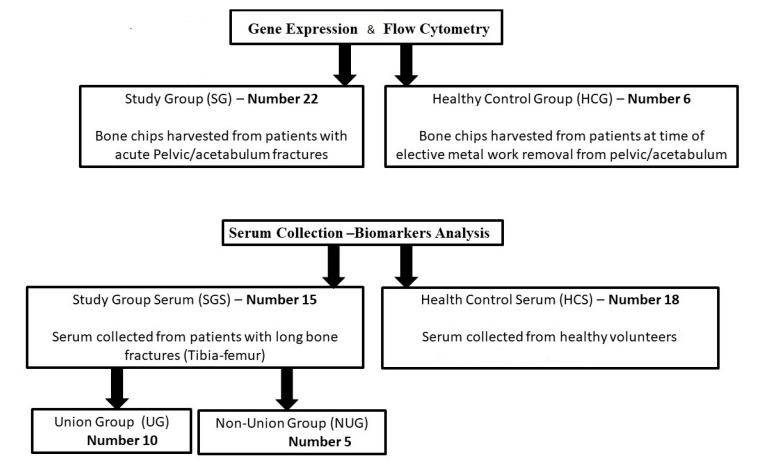
Schematics of the study groups used in this work. Of note, 4 samples from the SG group did not yield sufficient/good-quality RNA to be included in the gene expression study. Similarly, the number of cells recovered after collagenase digest was not sufficient to perform the flow cytometry analysis on all samples, leaving 4 HCS and 15 fractures.

**Figure 2:**
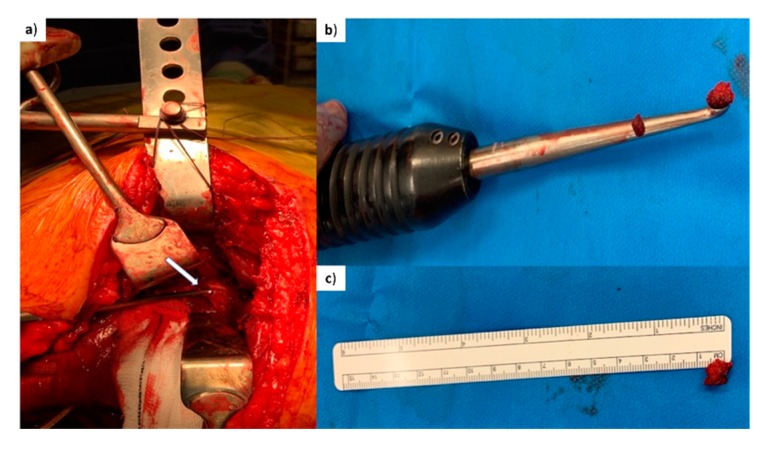
Surgical procedure. (**a**) Intra-operative picture demonstrating trabecular bone being harvested with a curette from the fracture edge of a posterior column fracture of pelvis (arrow points at the tip of the curette being inserted at the trabecular bone of the posterior column fracture for the harvesting of a small piece of bone). (**b**) Curette demonstrating the piece of trabecular bone (chip) harvested. (**c**) Piece of trabecular bone (before washing) placed next to a ruler (average 0.5 cm bone chip).

In order to standardize the anatomical location of tissue harvesting and to have the potential to harvest tissue with different length of delay since trauma, patients admitted with acute fractures of the pelvis and acetabulum requiring reconstruction were invited to participate in this study. This cohort of patients was found to be the ideal study group since our institution is a tertiary referral centre for pelvic and acetabulum injuries and due to transfer delays operative intervention occurs between 2 and 15 days after injury. Patients with pathological pelvic ring fractures undergoing reconstruction were excluded. During surgery, bone fragments were obtained from the fracture site (Figure 1, study group SG, *n* = 22). Following the surgical approach, the fracture area was directly visualised (pictures in supplementary material). Bone samples were taken from trabecular (cancellous) bone in the middle of the fracture, using a biopsy curette instrument (Figure 2). Samples were of the approximate same size/quantity in all cases (amount contained within the curettage volume area).

In addition, bone fragments were obtained at the time of elective surgery, during removal of metal work on fully healed bone from the pelvis in a control group including patients who had experienced a fracture, over 1 year before (healthy bone control group HCG, *n* = 6), using the same biopsy curette instrument. They had fully healed clinically and returned to their previous functional state. Therefore, we believe that all bone repairing physiological processes had been fully completed with no residual impact of the original fracture, allowing us to consider these as heathy bones.

Study was approved by research ethics committee (REC 06/Q1206/127) and all participants provided informed consent.

### 2.2. Patients for Serum Collection

Peripheral blood samples were collected in Serum Separator Tubes (Vacutainer) from adult patients (Study Group Serum, SGS = 15, under the same ethically approved project) treated for long bone isolated fractures (femur and tibia). After clotting for least 30 min, samples were centrifuged at 2000 × *g* for 15 min. Serum was aliquoted and stored at −80 °C until analysed. Samples were collected at day 1 (within 24 h of injury), and subsequently at days 3 and 5 following injury. The outcome recorded was non-union versus osseous fracture healing (Union). Non-union was defined as failure of the fracture to progress to healing radiographically with the presence of bridging callous on at least 3 cortices by a period of 9 months. Control serum was obtained from healthy volunteers (HCS, *n* = 18, from a biomarker study REC 09/H130798) who were not on any regular medication and had not suffered acute trauma or fractures within the past 24 months.

All participants signed an informed consent. Groups are summarised in Figure 1.

### 2.3. Gene Expression Profiling

Bone fragments from the fracture site/control bone were washed in saline solution and frozen at −80 °C for RNA extraction. Bone fragments were lysed in a guanidine-based buffer, followed by a phenol/chloroform isolation method. All RNA samples were treated with Ambion “DNA-free” kit for genomic-DNA removal. Concentrations and measures of the RNA quality were obtained using a Nanodrop spectrophotometer (ThermoFisher, Wilmington, DE, USA). 400 ng of RNA was subsequently converted into cDNA using a High-Capacity cDNA Reverse Transcription Kit (Applied Biosystems^®^, Loughborough, UK). Gene expression profiling was performed using Custom TaqMan Array (format 96a; both from Applied Biosystems). Exon-spanning, ‘3’ most’ TaqMan assays (96) were selected for the array when possible. The Ct values for the genes of interest were normalised to the endogenous control HPRT using the formula [ΔCt = Ct_target gene_ − Ct_housekeeping gene_], relative expression was calculated as 2^−ΔCt^ and used for analysis. Appropriate non template and non-reverse transcription controls were run alongside samples. Levels of expression of some genes were below detection and were arbitrary set at a threshold of detection of 0.001 (equivalent to the lowest detectable value by qPCR), to be compared to samples with detectable levels, notably comparing healthy bone homeostasis and then fracture samples.

### 2.4. Flow Cytometry

Bone fragments used for cell analysis were first manually minced using a rongeur, thoroughly washed with PBS and then placed in low-glucose modified Dulbecco’s eagle medium (Life Technologies, Paisley, UK) containing 20% fetal calf serum and collagenase (3000 units per gram of bone, Worthington Biochemical Corporation, NJ, USA) for 4 h at 37 °C as previously described. After completion of the bone digest, the fraction containing cell suspension was recovered. To recover the remaining cells, bone fragments were further washed with PBS which was added to the digest cell fraction. After pelleting at 100× *g* for 1 min to eliminate bone debris, supernatant was spun again at 300× *g* for 10 min to pellet cells.

For flow cytometry, cells were re-suspended in 30 µL of FACS buffer (PBS + 0.01% Sodium Azide) containing 1% Bovine serum albumin (as blocking agent) and incubated with varying combination of antibodies at 4 °C for 30 min (wrapped in aluminium foil). Cells were then washed with 200 µL cold FACS buffer. In total, 5 µL of live cell 7-aminoactinomycin D (7AAD) die (Abcam, Cambridge) were added and cells analysed by flow cytometry. Antibodies used were for Panel-1: anti-CD45, anti-CD3, anti-CD4, anti-CD8, anti-CD56, anti-CD19, anti-CD14 and for Panel-2: anti-CD45, anti-CD271, anti-CD90 and anti-CD73 (Table S1). All analyses were performed on LSRII 4 laser flow cytometer (BD-biosciences). Data analysis was done by using FACSDiva software (BD-biosciences).

### 2.5. Serum Markers

Candidates were measured using quantitative sandwich enzyme immunoassay technique following the manufacturer instructions. Commercially available human Quantikine High Sensitivity ELISA kits were used: Interleukin 8 (IL8), Placenta Growth Factor (PlGF), Tumour growth factor beta1 and 2 (TGF-beta1 and beta2), Monocyte chemoattractant protein 1 MCP-1, Vascular endothelial growth factor (VEGF), all from R&D systems (Biotechne, Abingdon, UK). The respective sensitivity of the kits for each molecule were as follows 0.4 pg/mL for IL8, 7 pg/mL for PlGF, 15.4 pg/mL for TGF-beta1, 7 pg/mL for TGF-beta 2, 10 pg/mL for MCP-1, and 5.0 pg/mL for VEGF.

### 2.6. Statistical Analysis

Differences between groups were analysed with the Mann–Whitney U test. Correlations were determined using linear Regression. Gene expression data were visualized as heat-map using Cluster v 3.0 and TreeView programs. Due to the high variability and low number of samples, no correction was applied either for gene expression or the cytokine analysis and the analysis is mostly indicative. Figures were drawn using GraphPad Prism software Version 8. Statistical analysis was performed using SPSS version 25.

## 3. Results

### 3.1. Patient Demographics for Bone Samples

Overall, 22 (seven female) consecutive patients with pelvic and acetabulum fractures consented to participate in this study (Figure 1, top panel) with a mean age of 45 years (range 20 to 66). Six individuals (three males and three females) formed the healthy pelvic control group (HCG), who were non-significantly younger (mean 42 years old, range 25–52), *p* > 0.05). Overall, out of the 28 patients recruited for this arm of the study, four were smokers (three from the study group and one from the healthy pelvic control group). None of the patients suffered from any comorbidity or were on medication that could have a negative impact on the fracture healing process. Moreover, there was no abnormal BMI index, mean BMI index 25.02 (range 19.8–28).

Samples obtained were prioritised for gene expression and then flow cytometry. All samples were used for the analysis. However, four did not yield a sufficient amount of RNA (quality or quantity) to be included in the gene expression analysis. In total, 24 bone samples were therefore used in the final dataset with 18 fractures (SG) and six controls (HCG).

Serum samples (Figure 1, bottom panel) were collected and stored from patients SGS (*n* = 15, 18–70 years of age) with long bone fracture at three time points (day of trauma (day 1), day 3 and day 5) and from healthy volunteers HCS (*n* = 18, age range 26 to 64 years, with 11 males and seven females). Outcome of the fracture (union versus non-union) was recorded over a 9-month period and used to classify patients.

### 3.2. Gene Expression in Healthy Bone at Homeostasis

We first analyse gene expression in six samples of healthy bone at homeostasis. Gene selected included transcript related to cell lineages, chemokines and their receptors, inflammation, growth factors and bone remodelling genes based on available literature (Table S2, including gene symbols and names).

**Figure 3:**
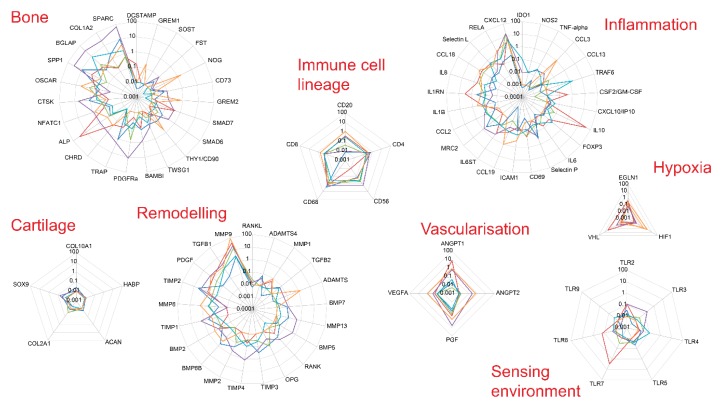
Gene expression levels in six biopsies from healthy bone. Gene expression levels were determined by qPCR. Genes were grouped by themes.

Altogether, there was large variability between the genes analysed and general trends for high and low expression in the different functional categories. Using transcript specific for immune cell lineage (Figure 2, immune cell lineage), we first established that there are lymphocytes of all lineages present in relatively small amounts in healthy bone. Inflammatory markers and immune response transcripts displayed low levels for most genes in all samples but with higher values for *CXCL12*, *RELA, Selectin-L, CCL18, IL1RN* and *IL1-beta* in three samples (Figure 3, Immune-related genes). Sensor genes for the environment were also expressed at low levels (*TLR* family) with hypoxia and angiogenesis genes being more variable in 2 samples. Cartilage related transcripts were mostly below detection or at very low levels. The bone related transcripts were expressed at low levels for half of the genes selected while higher levels were consistently detected for Osteonectin*/SPARC,* Osteocalcin*/BGLAP*, Osteopontin*/SPP1*, Collagen-1A2 *(COL1A2)*, *ALP* or catepsin-K*/CTSK*. Bone remodelling factors were showing a dominant *MMP9/TIMP2, PDFG* and *TGF-beta-*1 expression pattern with low levels of *BMPs*, *RANK/RANKL/OPG*.

**Figure 4:**
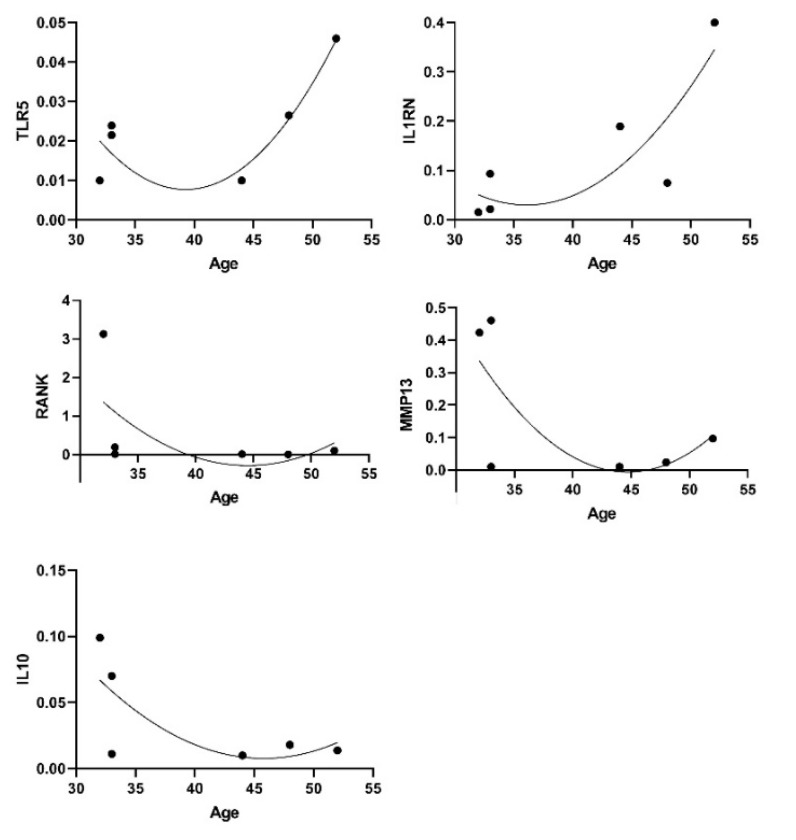
Genes and age associations.

We observed a few genes being expressed at higher levels in male (*CXCL10, NGFR,* cathepsin-K*/CSTK, MRC2, COL1A2, TIMP 3* and *4, TWSG1*) and some in females (*HIF1A, IL8* and *OSCAR*) although not significant due to small numbers *n* = 6. Considering our small number of healthy samples and the relatively small age range (25–52), positive trends for associations with age were observed for TLR5 and IL1RN while reduction was seen for *RANKL/TNFSF11, IL10, MMP13* (*n* = 6, −0.600 < rho < + 0.600, *p* < 0.05 although without correction for multiple testing) (Figure 4).

### 3.3. Gene Expression Profiling at the Fracture Site: Patients Clustering

**Figure 5:**
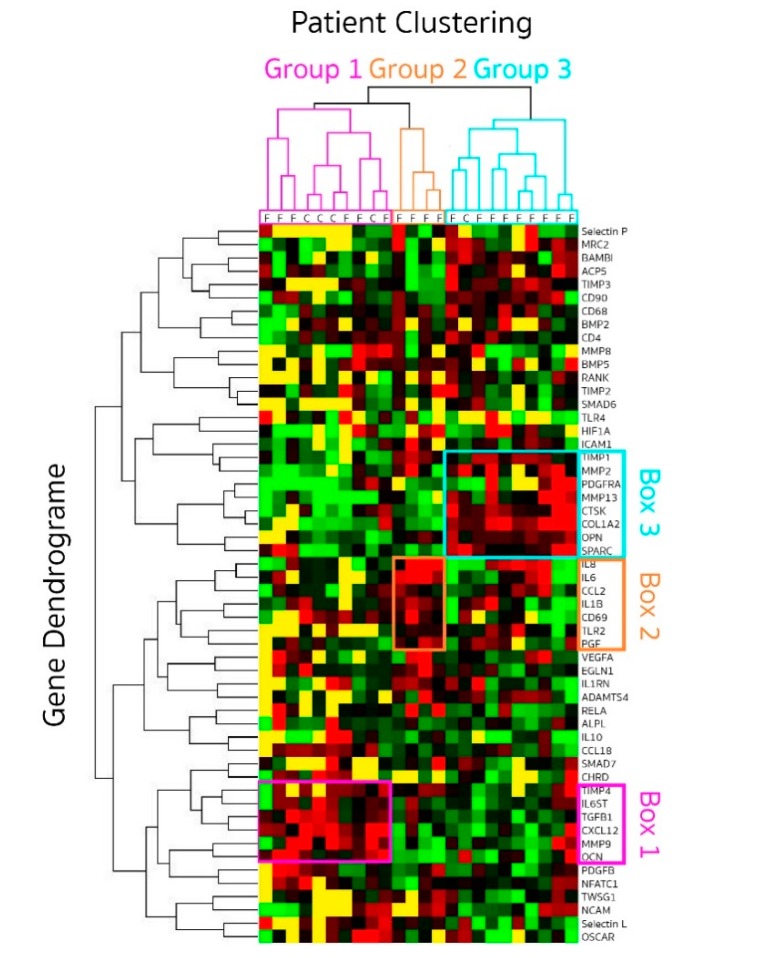
Gene expression profiling displayed as a heat map following hieratical clustering. Genes expression was quantified in bone genes from patients (SG *n* = 18) at different time points (2 to 14 days) after fracture and healthy controls (HCG *n* = 6). Clustering grouped patient in three groups (pink, blue and orange) displayed by the patient dendrogram at the top. Genes whose expressions were closely related are illustrated by the gene dendrogram on the left. Level of expression for each gene is coded red when high and green when low. Below detection levels are coded in yellow.

As previously stated, 24 samples were used in the final dataset with 18 fracture (SG) and six healthy control samples (HCG). To identify patterns of differential expression between control and fractured bone, an unbiased hierarchical clustering analysis was performed. To perform such analysis, we had to limit the number of genes used to those showing detectable levels in most samples. These criteria left 54 genes to be analysed. Results were displayed using a heat-map. Overall, based on this hieratical clustering approach, samples segregated in three groups (Figure 5): group 1, 10 samples (six fractures and four healthy controls—pink on the sample hierarchies); group 2, four samples (all fractures—orange); group 3, 10 samples (nine fractures and one healthy control—blue).

We investigated the difference between patients in each cluster for age, gender and time delay from fracture to surgery. No association could be detected with age or sex. The mean time delay between sustaining trauma and surgery for the patients in group 1 was 3.4 days (range 2–4), also grouping with four healthy control bone. In group 2, the time delay was longer with a mean time of 7 days (range 4–8) and even longer in group 3 being 9.3 days (range 8–14).

This data suggested a progression in the gene expression profile with time, short delays between trauma and surgery being more closely associated with healthy bone in group 1.

### 3.4. Gene Clustering

Three distinct sets of genes were up-regulated and driving the clustering of samples (Figure 5), either early (pink box 1 on the gene ID list for group 1) or late (blue box 3 for group 3) with a third set in the intermediate time group (orange box 2 for group 2).

The early gene set (box 1) included high expression levels (red on the heat-map) of *IL6ST*, *TGFbeta-1, CXCL12, MMP9, TIMP-4* and osteocalcin/*BGLAP*. This gene set suggests recruitment of immune and mesenchymal cells through high expression of *CXCL12* [20,21] and the possible responsiveness of these cells to interleukin-6 (*IL6ST* signalling sub-unit of the IL6R) and change in the balance of degradation enzymes and their inhibitors. As healthy bone (*n* = 5) clustered with the early fractures (*n* = 6), we analysed the difference between these 2 types of samples. Despite small numbers, the two main differences were reduced expression of osteocalcin/*BGLAP* (*p* = 0.019) but increased expression of *CXCL12* (*p* = 0.013) early after fractures. This is suggesting that an early signalling events in healing may be recruitment of lymphocytes and mesenchymal cells, changing the relative local composition of cell population. An early shift in the *MMP/TIMP* balance was also seen with trends for lower *MMP9* and higher *TIMP1* (both *p* = 0.099), as well as early reductions of *TGF-beta1* (*p* = 0.075). Other gene outside of box 1 also showed trends for recused expression for *OSCAR* (*p* = 0.051), and higher expression of *ANGPT2* (*p* = 0.073) may also suggest early involvement of angiogenesis.

The intermediate set of genes (box 2) included high expression levels of the inflammatory cytokines *IL1-beta, IL6* and *IL8*, the *CCL2* chemokine (also known as monocyte chemoattractant protein-1, *MCP1*), the T-cell activation marker *CD69*, the innate immunity receptor *TLR2* and vascularization with placental growth factor/*PGF*. *VEGF* also appeared more expressed in this group (3/4 patients). Box 2 genes were much less expressed in group 1 and group 3 (mostly low levels in green and below detection in yellow) with the exception of *IL8* in 5/9 patients and *IL6* in 2/9, which remained high (red) in group 3. Altogether, the increased expression of a marker of T-lymphocyte activation *CD69* and that of *TLR2*, a receptor that mediates immune response to Gram-positive bacteria appears to link this phase of the fracture repair process to an inflammatory process, highlighted by *IL1-beta*, *IL6* and *IL8* in this intermediate stage.

The late set of 7 genes (box 3) included high expression of osteonectin/*SPARC*, osteopontin/SPP1, platelet derived growth factor receptor alpha (*PDGFRA*), *MMP-2*, *MMP13*, *TIMP1*, *COL1A2* and cathepsin K*/CSTK*. These genes appear to be closely cooperating with each other towards the regulation of bone turnover. They were clearly at lower levels earlier in the repair process (group 1 and 2). In addition, 2 more genes (not include in the blue box) appeared upregulated in these samples: tartrate-resistant acid phosphatase 5 (TRAPS*/ACP5*) and *TIMP3*, which are also both involved in osteoclast activity. These genes are mainly related to bone signalling events and suggest that this may be the stage at which bone repair is initiated in human.

Altogether, these data suggest a progression in levels of expression of signalling molecules from cell recruitment (immune and mesenchymal) to inflammation and then initiation of repair process. We then examined gene expression levels for possible relationship between signalling molecules over the time delay between trauma and surgery (excluding the control bone samples). *TGF-beta1* and *SMAD7* (a transcription factor in the TGF signalling cascade) were correlated (rho= 0.707, *p* = 0.001) suggesting activation of this pathway. Similarly, *SMAD6* levels were related to those of bone morphogenetic protein 5 (*BMP5*, rho = 0.734, *p* = 0.007) and activin membrane-bound inhibitor (*BAMBI*, rho = 886, *p* = 0.001). Levels of expression of *CCL2* were related to the loss of collagen-10 expression (*COL10A1*, rho= −0.857 *p* = 0.014), higher *Gremlin-1* (rho = 0.811, *p* = 0.001), higher *TIMP1* (rho = 0.511, *p* = 0.0001) and higher *TNF-alpha* (rho = 0.800, *p* = 0.010). *IL8* weakly correlated with the loss of *RANK* (rho= −0.512, *p* = 0.043). These correlation suggest a timely process whereby signalling occurs early for *TGF-beta1* (high in group 1, quite low after), at an intermediate time for *CCL2* (only high in Group 2) and at a late time for *BAMBI* (only high in group 3) while *BMP5* expression do not seems to be time dependent and remain quite steady.

### 3.5. Immune and Mesenchymal Stem Cell Recruitment at Site of Fracture

**Figure 6:**
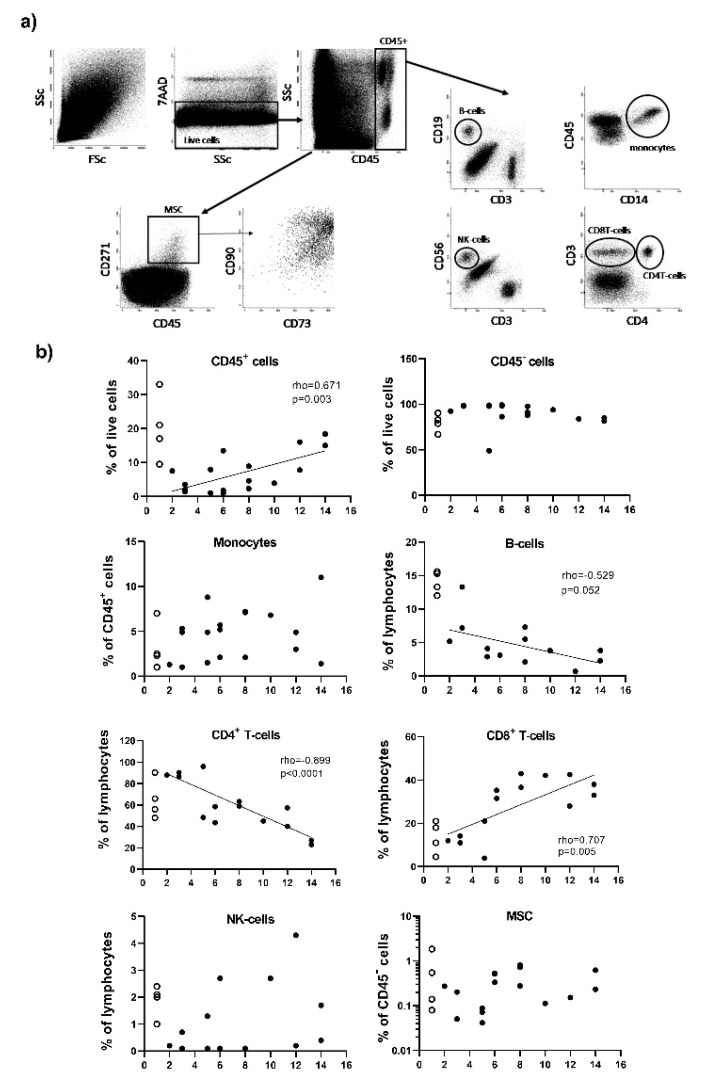
Flow cytometry analysis of the cells present in healthy bone and at the fracture site. (**a**) Flow cytometry gating strategy. In total, 100,000 events were recorded (1st plot FSc/SSc). Live cells were initially gated as 7AAD- events (2nd plot: SSc/7AAD). Doublet exclusion was performed (not displayed in figure). CD45+ and CD45– cells were then gated from the 3rd plot: (CD45/SSc). MSCs were then gated from the CD45- cell population using the expression of CD271 (plot CD45/CD271), also being CD90 and CD73 double positive (plot CD73/CD90 gated on CD271+ cells). Monocytes were directly gated from the CD45+ cells using a CD14/CD45 plot. Lymphocytes were gated from the CD45+ cells using 4 cell surface marker CD3, CD4, CD19 and CD56. Subsets of lymphocytes were identified as T-cells (CD3+CD4+ and CD3+CD4-), B-cell (CD19+) and NK-cells (CD56+). Total lymphocytes weres then calculated as the sum of events for each subset and each subset was reported as a % of total lymphocyte events. (**b**) Plots displaying frequency of cells with respect to time after trauma. Patients SG (*n* = 15) are represented by closed symbols and healthy bone HCG (*n* = 4) by open symbols (displayed at day 1 for convenience). Correlation with time were established for the fracture samples only (rho values displayed on the plot).

To further understand the timing of events in fracture healing, we analysed MSC and immune cell composition (Figure 6a) of healthy bone (*n* = 4, open symbols, displayed at day 1 for convenience in Figure 6b) and their recruitment at the fracture site (*n* = 15, closed symbols) with respect to the time delays since trauma.

In healthy bone, we observed relatively more immune cells (CD45+, % of total live cells) than non-hematopoietic cells (CD45- cells). This was associated with similar proportions of monocytes (CD14+ cells) and CD4+T-cells at early time post fracture (day 2), while there appear to be more CD8+T-cells and NK cells (CD56+) but less B-cells (CD19+). The frequency of MSC in healthy bone appears to be similar to early time post fracture, although in two or three samples only, with the last one showing 100 times more cells.

The total amount of immune cells (CD45+) kept increasing with time (Figure 6b, panel b, rho = 0.671, *p* = 0.003). Within the CD45+ immune cells, monocytes were not varying with time and represented on average 7% (+/−6%) of CD45+ cells. Lymphocytic cells represented 16% (+/−6.5%) of CD45+ cells and tended to increase with time (data not shown, rho = 0.547, *p* = 0.055). Within the lymphocytes, there was further change in the subsets present with increasing representation of CD8+T-cells (rho = 0.707, *p* = 0.005), but a reduction of CD4+T-cells (rho = −0.899 *p* = 0.0001), a trend for B-cells reduction (rho = −0.529, *p* = 0.052) and no change in CD56+NK-cells which were observed a lower frequency than in healthy bone. Contrary to CD45+ cells, non-hematopoietic cell (CD45-) did not vary with time delay from trauma to surgery. MSCs were quite rare (average 0.59% +/−0.87%) and did not show particular tends for recruitment to the site of fracture.

### 3.6. Serum Markers

Certain gene transcripts expressed during healing may also be detectable as circulating proteins. Consequently, we explored the levels of expression of six soluble molecules in serum samples from patients for whom a union (U) versus non-union (NU) outcome was already known. Candidates were selected on the basis of being overexpressed at any point during fracture healing and included IL8, CCL2/MCP-1, PlGF, TGF-beta1, TGF-beta2 and VEGF.

**Figure 7:**
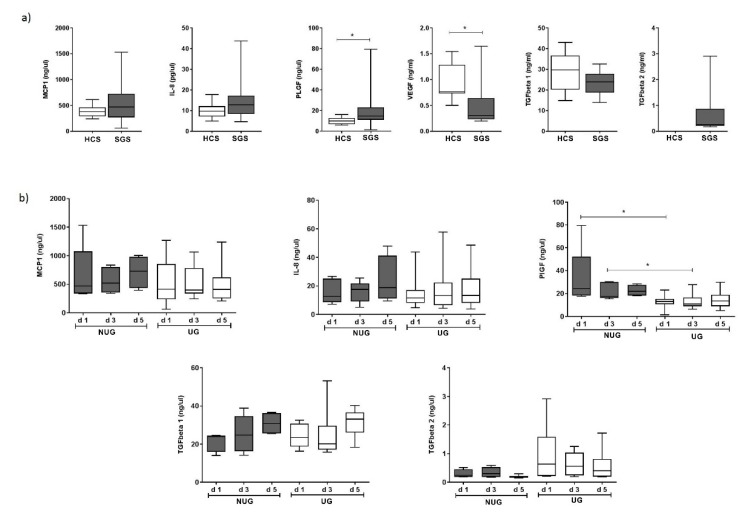
Circulating levels of soluble factors: (**a**) Levels were compared between HCS and 15 patients SGS with a long bone fracture at day 1 post trauma. (**b**) Levels of five factors were compared between patients with a long bone fracture with a union (UG, *n* = 10) or non-union (NUG, *n* = 5) outcome at day 1, 3 and 5 after trauma. * Significant difference (*p* < 0.05).

We first compared levels of these molecules between patients with long bone fractures (day 1) and healthy controls (Figure 7a). Within 24 h of fracture, patient SGS (*n* = 15) compared to HCS (*n* = 18) showed higher levels of IL8, MCP-1 and PlGF (Figure 7a), reaching significance for PlGF (*p* = 0.003). Levels of TGF-1 and VGF appeared lower in fracture patients compared to HCS, reaching significance for VGF (*p* = 0.006). We did not have sufficient volume of serum to test TGF-beta2 in HCS. Reported levels by manufacturers average at 400 up/mL (*n* = 63) suggesting that levels were probably increased in patients although we could not verify this directly.

We then compared UG versus NUG over 5 days (Figure 7b). Due to the small number of patients in the NU group (*n* = 5), this analysis is only indicative. Levels of MCP-1 and IL8 showed no clear trend over a 5-day period in both NUG and UG and did not appeared different at any point. Levels of and TGF-beta 1 increased over 5 days, but with no difference between UG and NUG. Levels of TGF-beta2 remained relatively stable over 5 days and appeared to be higher in UG although non-significantly (*p* = 0.195). The main clear difference between UG and NUG was observed at baseline and day 3 for levels of PlGF which were higher in NUG patients (*p* = 0.003 at day 1 and *p* = 0.019 at day 3) while the difference remained, but was less marked at day 5 (*p* = 0.0999).

## 4. Discussion

To our knowledge, the herein study is the first to explore the dynamics of gene expression in bone at homeostasis in healthy, uninjured volunteers. Using gene expression analysis, we investigated gene profiles in healthy bone, which interestingly, was clearly perturbed post-fracture. Exploiting gene expression data, we investigated soluble molecules that could be detected in the circulation, as biomarkers of fracture non-union and showed that PlGF levels are significantly associated with fracture outcome.

In healthy bone, we observed low expression levels of immune cell lineage marker, low levels of inflammation markers (cytokines), high levels of bone protein constituents such as osteonectin/*SPARC* (secreted extracellular matrix protein, vital for mineralisation) with an additional role in angiogenesis [22], osteocalcin/*BGLAP* (calcium binding protein regulating mineralisation in bone), osteopontin/*SPP1* (secreted structural protein) supporting bone by anchoring osteoclasts to the mineral matrix of bones [23] with additional role in immune cell recruitment and cytokine expression [24,25], *Collagen-1A2* (providing strength to the bone tissue), as well as very low levels of cartilage protein. Data also suggested a balance between pro- and anti-bone signalling factor with high PDGF (a blood vessel stabiliser and growth factor for mesenchymal (stem) cells) and *TGF-beta1* (supporting proliferation/differentiation of cells) with a role in fracture [26] but low BMPs (inducer of bone formation), *RANK/RANKL/OPG* (regulators of osteoclast activity) as well as balanced between degrading enzymes (mainly *MMP9* here) and their inhibitors (*TIMP2*).

Post-fracture, this homeostasis is clearly perturbed, and our data are the first to demonstrate changes in gene expression over time during fracture healing. We observed that the bone repair process starts by a phase of cell recruitment, during which gene expression confirmed the early expression of chemokine (*CXCL12*) attracting immune cells (confirmed by flow cytometry data) and potentially MSC [27] towards forming a soft callus. At that stage, the balance between degrading enzymes and their inhibitor is also tilted towards degradation (*MMP9/TIMP4*), which is an important process to allow invasion of new blood vessels [27]. This phase is followed by inflammation (IL1b, IL6, IL8), more recruitment (*CCL2*) and suggestion of immune cell activation (*CD69*) as well as high *TLR2* expression. During this phase, the expression of *PlGF* (a homologue to *VEGF* [28]) previously associated with fracture healing in an animal model [29,30] is increased. Hardening of the callus may then start (late stage group), with the higher expression of bone matrix proteins (osteonectin/*SPARC,* osteopontin/SPP1, collagen-1A2), as well as cathepsin-K/*CTSK*, TRAPs/*ACP5* and TIM3), while tissue degradation is still active (MMP-2, MMP13). The longest time delay from fracture in the samples we obtained may not have allowed us to fully observe this late bone repair phase in human, notably as Collagen-10 was under/not yet detected, although cathepsin-K/*CTSK* (a protein released from mature osteoclasts and involved in cartilaginous and mineralised callus remodelling [31] and *MMP2/13* (considered to be repair factors as reviewed in [32,33], showed increased expression at this late stage.

The molecular signals governing bone repair have been previously investigated in animal models [2,34]. Stages in animals are usually described as (i) inflammatory, (ii) soft callus, (iii) hard callus and (iv) remodelling. The early stage (inflammation), was marked by the early increase (then reduction) of *IL1b, IL6, TNF-a*, hypoxia, recruitment of progenitor stromal cells [2,34]. Our data in humans are well fully aligned to these, notably in terms of an increase in *IL1* and IL6 (but not *TNF-alpha*) followed by their reduction (but an increase in *TNF-alpha*). Nonetheless, the second series of events in humans (intermediate stage) is the recruitment of immune cells that is delayed by a few days (day 5 to 8) in humans compared to mice.

Recruitment of CD8 and loss of regulatory CD4 T-cells was also observed early in animal models (over the initial 3 days) [34], showing similar pattern for human CD8 recruitment, although more slowly (and up to day 12) while FoxP3 expression (a marker of CD4 regulatory cells) was detectable in healthy bone and late fracture suggesting an early loss (like in animal) and recovery later. The induction of vascularisation is more controversial in animal model, and was showed in several phases [34] (inflammatory, soft and hard callus formation), or only late (expression of *ANG-1* and *VEGF* after inflammation phase) [2]. In human, there are no published data in trabecular bone to our knowledge. Studies in fracture hematoma samples equivalent of a T0 (no fracture) [35], showed similar percentage of CD14+ monocytes (approximately 5% of CD45+ cells), and amongst lymphocytes similarly 20% of B-cell, 12% of CD8 T-cells but only 23% of CD4+T-cells, while we observed approximately 60%. Within 72 h of the fracture, there was also no change in monocytes, loss of B-cells, increase in CD8+T-cells and limited change in CD4+T-cell subsets at this early time [35]. Similar to our data, increased IL1-beta, IL6 and IL8 (protein levels) were detected early in hematoma [35] while we did not see a clear increase in IL10, IL1R or VEGF early (although at mRNA levels rather than protein). In hematoma (72 h of fracture) from patients with autoimmune conditions, exaggerated expression of the same factor was also reported [36] as well as increased in transcript for osteopontin/*SPP1* and *HIF1* [37].

This is different in humans, with a massive reduction in *ANGPT1* over the overall period studied (0–15 days), while *ANGPT*2 did not show variation (similar levels to heathy bone). VEGF (like in animals) and *PGF* (reported here for the 1st time) showed increased expression only at the late stage. Both of these pathways are believed to be upregulated during bone repair [38] *VEGFs* by promoting neo-angiogenesis while, angiopoietin 1 and 2 support the formation of larger vessel structures and the development of collateral branches from existing vessels [39] or as shown more recently, via an autophagy-related mechanism [40]. Other growth factors such as *PDGF* and the *TGF-betas* were reported in late stages in animals (hard callus) and showed similar steady low increase in expression over time in humans for *TGF-beta2* (no change in *TGF-beta1*) but a reduction followed by increase for *PDGF*. A hard callus formation with mineralisation of the tissue was not really observed in our human samples although an increase in the expression of *TGF-beta2* and *BMP8* suggest it may be initiated at that stage.

Due to limited amount of serum we could not test all potential soluble factor candidates (for example *CXCL12* or *MMPs/TIMPs*). Out of the 6 proteins tested, we observed clear potential for PlGF as an early indicator of NU within 24 h of fracture. Despite the small number of samples available, the absence of overlap in the distribution between UG/NUG at day 1 post-fracture should make it possible to define a cut-off to be applied for any prediction algorithm in the future and test the clinical utility of measuring PlGF in larger number of patients. TGF-beta2 also appears interesting, this time with high levels being associated with Union. Therefore, a ratio of the 2 candidates may prove quite powerful in a prediction model. TGF-beta 1 results were disappointing as it was previously associated with NUG in humans [26,41] but it remained mechanistically important for the healing process, with increasing levels being observed, although both in UG/NUG. VEGF levels were lower in fracture samples at day 1 in contrast to previous results [42].

Placental growth factor was previously associated with fracture healing in an animal model [29,30]. Knock-out of *PGF* showed a lack of bone formation (with cartilage accumulation) while it was also essential for the recruitment of inflammatory cells and early vascularisation of the fracture. The absence of osteogenic stimuli in the knock-out was further investigated and *PlGF* was shown to have direct effect on the proliferation and osteogenic differentiation of MSCs [30]. Serum levels in UG patients showed a steady state increase over 5 days, like in mice, indicating that PlGF is continuously increasing over the repair process. In contrast, in NUG, a massive surge in PlGF levels was observed followed by a rapid decline. Further investigations of this particular growth factor are needed to understand its fine-tuning role in human fracture repair, as well as its potential as biomarker of Non-Union.

While this study is unique for its recruitment and novel findings in human, it has some limitations. Firstly, it could be argued that the number of patients is small. However, with the implementation of major trauma centres allowing most patients to be operated early nowadays, our study subjects were uniquely, consecutive patients with bone biopsies at the fracture site taken at different time points until surgery. Secondly, although a comprehensive list of 94 genes (+2 housekeeping genes) was tested, we may have missed important/unknown factors in the healing process. Our data are therefore only as good as the gene selected were relevant. Thirdly, the biomarker study carried out was limited by the number of patients recruited but definitely provided a platform for a more comprehensive study of selected candidates in the future.

## 5. Conclusions

In conclusion, this is the first study in humans to describe the dynamics of gene expression after fracture as well as at homeostasis in healthy bone. Exploiting gene expression data, soluble molecules were investigated as potential biomarkers of fracture non-union in the peripheral circulation. We found that PlGF levels were significantly associated with fracture outcome. The herein data can form the basis for the design of future studies investigating the cascade of events following fractures and inform further studies of predictor of fracture non-union.

## Supplementary Materials

The following are available online at https://www.mdpi.com/2077-0383/9/2/492/s1, Table S1: Antibody details; Table S2: List of preselected genes with assays IDs used for qPCR.

## References

[1] Giannoudis P.V., Einhorn T.A., Marsh D. (2007). Fracture healing: The diamond concept. Injury.

[2] Dimitriou R., Tsiridis E., Giannoudis P.V. (2005). Current concepts of molecular aspects of bone healing. Injury.

[3] Augat P., Faschingbauer M., Seide K., Tobita K., Callary S.A., Solomon L.B., Holstein J.H. (2014). Biomechanical methods for the assessment of fracture repair. Injury.

[4] Ekegren C., Edwards E., de Steiger R., Gabbe B. (2018). Incidence, costs and predictors of non-union, delayed union and mal-union following long bone fracture. Int. J. Environ. Res. Public Health.

[5] Gómez-Barrena E., Rosset P., Lozano D., Stanovici J., Ermthaller C., Gerbhard F. (2015). Bone fracture healing: Cell therapy in delayed unions and nonunions. Bone.

[6] Panteli M., Pountos I., Jones E., Giannoudis P.V. (2015). Biological and molecular profile of fracture non-union tissue: Current insights. J. Cell. Mol. Med..

[7] Lee S.H., Choi Y. (2015). Communication between the skeletal and immune systems. Osteoporos. Sarcopenia.

[8] Schmidt-Bleek K., Schell H., Kolar P., Pfaff M., Perka C., Buttgereit F., Duda G., Lienau J. (2009). Cellular composition of the initial fracture hematoma compared to a muscle hematoma: A study in sheep. J. Orthop. Res..

[9] Kovtun A., Messerer D.A., Scharffetter-Kochanek K., Huber-Lang M., Ignatius A. (2018). Neutrophils in tissue trauma of the skin, bone, and lung: Two sides of the same coin. J. Immunol. Res..

[10] Baht G.S., Vi L., Alman B.A. (2018). The role of the immune cells in fracture healing. Curr. Osteoporos. Rep..

[11] Marsell R., Einhorn T.A. (2011). The biology of fracture healing. Injury.

[12] Edderkaoui B. (2017). Potential role of chemokines in fracture repair. Front. Endocrinol..

[13] Cheung R.K., Utz P.J. (2012). Correction: Screening: Cytof—The next generation of cell detection. Nat. Rev. Rheumatol..

[14] Takayanagi H. (2005). Mechanistic insight into osteoclast differentiation in osteoimmunology. J. Mol. Med..

[15] Zaiss M.M., Axmann R., Zwerina J., Polzer K., Gückel E., Skapenko A., Schulze-Koops H., Horwood N., Cope A., Schett G. (2007). Treg cells suppress osteoclast formation: A new link between the immune system and bone. Arthritis Rheum..

[16] Kolar P., Schmidt-Bleek K., Schell H., Gaber T., Toben D., Schmidmaier G., Perka C., Buttgereit F., Duda G.N. (2010). The early fracture hematoma and its potential role in fracture healing. Tissue Eng. Part B Rev..

[17] Colburn N.T., Zaal K.J., Wang F., Tuan R.S. (2009). A role for γ/δ t cells in a mouse model of fracture healing. Arthritis Rheum..

[18] Claes L., Recknagel S., Ignatius A. (2012). Fracture healing under healthy and inflammatory conditions. Nat. Rev. Rheumatol..

[19] Harvey E.J., Giannoudis P.V., Martineau P.A., Lansdowne J.L., Dimitriou R., Moriarty T.F., Richards R.G. (2011). Preclinical animal models in trauma research. J. Orthop. Trauma.

[20] Xing J., Hou T., Jin H., Luo F., Change Z., Li Z., Xie Z., Xu J. (2014). Inflammatory microenvironment changes the secretory profile of mesenchymal stem cells to recruit mesenchymal stem cells. Cell. Physiol. Biochem..

[21] Churchman S.M., Ponchel F., Boxall S.A., Cuthbert R., Kouroupis D., Roshdy T., Giannoudis P.V., Emery P., McGonagle D., Jones E.A. (2012). Transcriptional profile of native cd271+ multipotential stromal cells: Evidence for multiple fates, with prominent osteogenic and wnt pathway signaling activity. Arthritis Rheum..

[22] Guweidhi A., Kleeff J., Adwan H., Giese N.A., Wente M.N., Giese T., Büchler M.W., Berger M.R., Friess H. (2005). Osteonectin influences growth and invasion of pancreatic cancer cells. Ann. Surg..

[23] Reinholt F.P., Hultenby K., Oldberg A., Heinegård D. (1990). Osteopontin—A possible anchor of osteoclasts to bone. Proc. Natl. Acad. Sci. USA.

[24] Chabas D., Baranzini S.E., Mitchell D., Bernard C.C., Rittling S.R., Denhardt D.T., Sobel R.A., Lock C., Karpuj M., Pedotti R. (2001). The influence of the proinflammatory cytokine, osteopontin, on autoimmune demyelinating disease. Science.

[25] Higuchi Y., Tamura Y., Uchida T., Matsuura K., Hijiya N., Yamamoto S. (2004). The roles of soluble osteopontin using osteopontin-transgenic mice In Vivo: Proliferation of cd4+ t lymphocytes and the enhancement of cell-mediated immune responses. Pathobiol. J. Immunopathol. Mol. Cell. Biol..

[26] Wildemann B., Schmidmaier G., Brenner N., Hüning M., Stange R., Haas N., Raschke M. (2004). Quantification, localization, and expression of igf-i and tgf-β1 during growth factor-stimulated fracture healing. Calcif. Tissue Int..

[27] Cuthbert R.J., Churchman S.M., Tan H.B., McGonagle D., Jones E., Giannoudis P.V. (2013). Induced periosteum a complex cellular scaffold for the treatment of large bone defects. Bone.

[28] De Falco S.J.E. (2012). The discovery of placenta growth factor and its biological activity. Exp. Mol. Med..

[29] Maes C., Coenegrachts L., Stockmans I., Daci E., Luttun A., Petryk A., Gopalakrishnan R., Moermans K., Smets N., Verfaillie C.M. (2006). Placental growth factor mediates mesenchymal cell development, cartilage turnover, and bone remodeling during fracture repair. J. Clin. Investig..

[30] McCoy R.J., Widaa A., Watters K.M., Wuerstle M., Stallings R.L., Duffy G.P., O’Brien F.J. (2013). Orchestrating osteogenic differentiation of mesenchymal stem cells—Identification of placental growth factor as a mechanosensitive gene with a pro-osteogenic role. Stem Cells.

[31] Soung do Y., Gentile M.A., Duong L.T., Drissi H. (2013). Effects of pharmacological inhibition of cathepsin k on fracture repair in mice. Bone.

[32] Henle P., Zimmermann G., Weiss S. (2005). Matrix metalloproteinases and failed fracture healing. Bone.

[33] Lieu S., Hansen E., Dedini R., Behonick D., Werb Z., Miclau T., Marcucio R., Colnot C. (2011). Impaired remodeling phase of fracture repair in the absence of matrix metalloproteinase-2. Dis. Model. Mech..

[34] Schmidt-Bleek K., Kwee B.J., Mooney D.J., Duda G.N. (2015). Boon and bane of inflammation in bone tissue regeneration and its link with angiogenesis. J. Tissue Eng. Part B Rev..

[35] Hoff P., Gaber T., Strehl C., Schmidt-Bleek K., Lang A., Huscher D., Burmester G.R., Schmidmaier G., Perka C., Duda G.N. (2016). Immunological characterization of the early human fracture hematoma. Immunol. Res..

[36] Hoff P., Gaber T., Strehl C., Jakstadt M., Hoff H., Schmidt-Bleek K., Lang A., Rohner E., Huscher D., Matziolis G. (2017). A pronounced inflammatory activity characterizes the early fracture healing phase in immunologically restricted patients. Int. J. Mol. Sci..

[37] Hoff P., Gaber T., Schmidt-Bleek K., Senturk U., Tran C.L., Blankenstein K., Lutkecosmann S., Bredahl J., Schuler H.J., Simon P. (2011). Immunologically restricted patients exhibit a pronounced inflammation and inadequate response to hypoxia in fracture hematomas. Immunol. Res..

[38] Gerstenfeld L.C., Cullinane D.M., Barnes G.L., Graves D.T., Einhorn T.A. (2003). Fracture healing as a post-natal developmental process: Molecular, spatial, and temporal aspects of its regulation. J. Cell. Biochem..

[39] Portal-Nunez S., Lozano D., Esbrit P. (2012). Role of angiogenesis on bone formation. Histol. Histopathol..

[40] Yin J., Gong G., Sun C., Yin Z., Zhu C., Wang B., Hu Q., Zhu Y., Liu X. (2018). Angiopoietin 2 promotes angiogenesis in tissue-engineered bone and improves repair of bone defects by inducing autophagy. Biomed. Pharmacother..

[41] Sarahrudi K., Thomas A., Mousavi M., Kaiser G., Köttstorfer J., Kecht M., Hajdu S., Aharinejad S. (2011). Elevated transforming growth factor-beta 1 (tgf-β1) levels in human fracture healing. Injury.

[42] Sarahrudi K., Thomas A., Braunsteiner T., Wolf H., Vécsei V., Aharinejad S. (2009). Vegf serum concentrations in patients with long bone fractures: A comparison between impaired and normal fracture healing. J. Orthop. Res..

