# Supplementary material for: Dynamics of Early Signalling Events during Fracture Healing and Potential Serum Biomarkers of Fracture Non-Union in Humans
Source: J Clin Med. 2020 Feb 11;9(2):492. doi: 10.3390/jcm9020492 (PMC7073571; doi:10.3390/jcm9020492)
Supplement: Supplementary file 1 [file jcm-09-00492-s001.pdf]

Supplementary material:

Dynamics of early signalling events during fracture healing and potential serum biomarkers of fracture non-union in humans.

Agata N Burska[1], Peter V Giannoudis[1,2*], Boon Hiang Tan[1,2], Dragos Ilas[1], Elena Jones[1] and Frederique Ponchel[1]

[1] Leeds Institute of Rheumatic and Musculoskeletal Medicine, University of Leeds, Leeds, UK

[2] Leeds Biomedical Research Centre, Leeds Teaching Hospitals NHS Trust (LTHT), Leeds, UK

* Correspondence: **P.Giannoudis@leeds.ac.uk**

Table 1. Antibody clones details.

marker	colour	Manufacturer	Cat number
**CD3**	FITC	AbD Serotec	MCA2184F
**CD4**	APC-Cy7	BD Biosciences	341115
**CD14**	Alex Fluor 700	BD Pharmingen	557923
**CD19**	Pacific blue	AbD Serotec	MCA1940PB
**CD56**	PE	BD Pharmingen	555516
**CD45**	PE-Cy7	BD Biosciences	557748)
**7AAD**		BD Pharmingen	559925

Table 2. List of preselected genes with assays IDs used for qPCR.

**ne Symbol / assay number**	**Gene full name (alternative name)**
ACAN-Hs00153936_m1	aggrecan
ACP5-Hs00356261_m1	acid phosphatase 5, tartrate resistant
DAMTS4-Hs00192708_m1	ADAM metallopeptidase with thrombospondin type 1 motif, 4
DAMTS5-Hs01095524_m1	ADAM metallopeptidase with thrombospondin type 1 motif, 5
ALPL-Hs00758162_m1	alkaline phosphatase, liver/bone/kidney
NGPT1-Hs00181613_m1	angiopoietin 1
NGPT2-Hs01048042_m1	angiopoietin 2
AMBI-Hs03044164_m1	BMP and activin membrane-bound inhibitor
BGLAP-Hs01587814_g1	bone gamma-carboxyglutamate (gla) protein; Osteocalcin (OCN)
BMP2-Hs00154192_m1	bone morphogenetic protein 2
BMP5-Hs00234930_m1	bone morphogenetic protein 5
BMP7-Hs00233477_m1	bone morphogenetic protein 7
BMP8B-Hs01629120_s1	bone morphogenetic protein 8b
CCL13-Hs00234646_m1	chemokine (C-C motif) ligand 13
CCL18-Hs04194943_s1	chemokine (C-C motif) ligand 18
CCL19-Hs00171149_m1	chemokine (C-C motif) ligand 19
CCL2-Hs00234140_m1	chemokine (C-C motif) ligand 2
CCL3-Hs04194942_s1	chemokine (C-C motif) ligand 3
CD4-Hs00181217_m1	CD4 molecule
CD68-Hs02836816_g1	CD68 molecule
CD69-Hs00934033_m1	CD69 molecule
CD8A-Hs00233520_m1	CD8a molecule
CHRD-Hs01000641_m1	chordin
OL10A1-Hs00166657_m1	collagen, type X, alpha 1
OL1A2-Hs01028971_m1	collagen, type I, alpha 2
OL2A1-Hs00264051_m1	collagen, type II, alpha 1
CSF2-Hs99999044_m1	colony stimulating factor 2 (granulocyte-macrophage); Granulocyte-Macrophage Colony-Stimulating Factor (GMCS
CTSK-Hs00166156_m1	cathepsin K
XCL10-Hs01124251_g1	chemokine (C-X-C motif) ligand 10
XCL12-Hs00171022_m1	chemokine (C-X-C motif) ligand 12
STAMP-Hs00229255_m1	dendrocyte expressed seven transmembrane protein
GLN1-Hs00254392_m1	egl-9 family hypoxia-inducible factor 1
OXP3-Hs01085832_m1	forkhead box P3
FST-Hs00246256_m1	follistatin
APDH-Hs99999905_m1	glyceraldehyde-3-phosphate dehydrogenase, **house keeping gene**
GREM1-Hs01879841_s1	gremlin 1, DAN family BMP antagonist
GREM2-Hs03986140_s1	gremlin 2, DAN family BMP antagonist
HIF1A-Hs00936371_m1	hypoxia inducible factor 1, alpha subunit
HPRT1-Hs99999909_m1	hypoxanthine phosphoribosyltransferase 1, **house keeping gene**
CAM1-Hs00164932_m1	intercellular adhesion molecule 1
IDO1-Hs00984148_m1	indoleamine 2,3-dioxygenase 1

IL10-Hs00961622_m1	interleukin 10
IL1B-Hs01555410_m1	interleukin 1, beta
L1RN-Hs00893626_m1	interleukin 1 receptor antagonist
IL6-Hs00985639_m1	interleukin 6 (interferon, beta 2)
L6ST-Hs00174360_m1	interleukin 6 signal transducer (gp130)
IL8-Hs00174103_m1	interleukin 8
MMP13-Hs00942589_m1	matrix metallopeptidase 13 (collagenase 3)
MMP1-Hs00899658_m1	matrix metallopeptidase 1 (interstitial collagenase)
MMP2-Hs01548727_m1	matrix metallopeptidase 2 (gelatinase A, type IV collagenase)
MMP8-Hs01029057_m1	matrix metallopeptidase 8 (neutrophil collagenase)
MMP9-Hs00957562_m1	matrix metallopeptidase 9 (gelatinase B, type IV collagenase)
MRC2-Hs00195862_m1	mannose receptor, C type 2; (CD280)
MS4A1-Hs00544819_m1	membrane-spanning 4-domains, subfamily A, member 1; (CD20)
CAM1-Hs00941833_m1	neural cell adhesion molecule 1; (CD56)
FATC1-Hs00542678_m1	nuclear factor of activated T-cells
NGFR-Hs00609977_m1	nerve growth factor receptor
NOG-Hs00271352_s1	noggin
NOS2-Hs01075529_m1	nitric oxide synthase 2, inducible
NT5E-Hs00159686_m1	5'-nucleotidase, ecto; (CD73)
OSCAR-Hs01100185_m1	osteoclast associated, immunoglobulin-like receptor
DGFB-Hs00966522_m1	platelet-derived growth factor beta polypeptide
DGFRA-Hs00998018_m1	platelet-derived growth factor receptor, alpha polypeptide
PlGF-Hs01119262_m1	placental growth factor; (PGF; PLGF)
RELA-Hs01042010_m1	v-rel avian reticuloendotheliosis viral oncogene homolog A; (P65)
SELL-Hs01053460_m1	selectin L
SELP-Hs00927901_m1	selectin P, CD62
MAD6-Hs00178579_m1	SMAD family member 6
MAD7-Hs00998193_m1	SMAD family member 7
SOST-Hs00228830_m1	sclerostin
SOX9-Hs01001343_g1	SRY (sex determining region Y)-box 9
PARC-Hs00234160_m1	secreted protein, acidic, cysteine-rich, Osteonectin (ON)
SPP1-Hs00959010_m1	secreted phosphoprotein 1, Osteopontin (OPN)
GFB1-Hs00998133_m1	transforming growth factor, beta 1
GFB2-Hs00234244_m1	transforming growth factor, beta 2
THY1-Hs00264235_s1	Thy-1 cell surface antigen; (CD90)
TIMP1-Hs00171558_m1	metallopeptidase inhibitor 1
TIMP2-Hs01091319_m1	metallopeptidase inhibitor 2
TIMP3-Hs00927214_m1	metallopeptidase inhibitor 3
TIMP4-Hs00162784_m1	metallopeptidase inhibitor 4
TLR2-Hs00610101_m1	toll-like receptor 2
TLR3-Hs01551078_m1	toll-like receptor 3
TLR4-Hs01060206_m1	toll-like receptor 4
TLR5-Hs01019558_m1	toll-like receptor 5
TLR7-Hs00152971_m1	toll-like receptor 7
TLR8-Hs00152972_m1	toll-like receptor 8
TLR9-Hs00152973_m1	toll-like receptor 9
NFAIP6-Hs01113602_m1	tumor necrosis factor, alpha-induced protein 6
TNF-Hs99999043_m1	tumor necrosis factor
FRSF11A-Hs00921372_m1	tumor necrosis factor receptor superfamily, member 11a; Receptor Activator Of Nuclear Factor Kappa B (RANK)
FRSF11B-Hs00900360_m1	tumor necrosis factor receptor superfamily, member 11b; Osteoprotegerin (OPG)
NFSF11-Hs01092186_m1	tumor necrosis factor (ligand) superfamily, member 11; Receptor Activator Of Nuclear Factor Kappa B Ligand (RANK
RAF6-Hs00377558_m1	TNF receptor-associated factor 6; Interleukin-1 Signal Transducer
WSG1-Hs00221028_m1	twisted gastrulation homolog 1
EGFA-Hs00900058_m1	vascular endothelial growth factor A
VHL-Hs01650959_m1	von Hippel-Lindau tumor suppressor
